# A novel knowledge-based prediction model for estimating an initial equivalent uniform dose in semi-auto-planning for cervical cancer

**DOI:** 10.1186/s13014-022-02120-4

**Published:** 2022-08-29

**Authors:** Cheng Tao, Bo Liu, Chengqiang Li, Jian Zhu, Yong Yin, Jie Lu

**Affiliations:** Department of Radiation Oncology, Shandong Cancer Hospital and Institute, Shandong First Medical University and Shandong Academy of Medical Sciences, No. 440, Jiyan Road, Jinan, 250117 China

**Keywords:** Equivalent uniform length, Generalized equivalent uniform dose, Knowledge-based prediction model, Auto planning

## Abstract

**Background:**

We developed a novel concept, equivalent uniform length (EUL), to describe the relationship between the generalized equivalent uniform dose (EUD) and the geometric anatomy around a tumor target. By correlating EUL with EUD, we established two EUD–EUL knowledge-based (EEKB) prediction models for the bladder and rectum that predict initial EUD values for generating quality treatment plans.

**Methods:**

EUL metrics for the rectum and bladder were extracted and collected from the intensity-modulated radiotherapy therapy (IMRT) plans of 60 patients with cervical cancer. The two EEKB prediction models were built using linear regression to establish the relationships between EUL_r_ and EUD_r_ (EUL and EUD of rectum) and EUL_b_, and EUD_b_ (EUL and EUD of bladder), respectively. The EE plans were optimized by incorporating the predicted initial EUD parameters for the rectum and bladder with the conventional pinnacle auto-planning (PAP) initial dose parameters for other organs. The efficiency of the predicted initial EUD values were then evaluated by comparing the consistency and quality of the EE plans, PAP plans (based on default PAP initial parameters), and manual plans (designed manually by different dosimetrists) for a sample of 20 patients.

**Results:**

Linear regression analyses showed a significant correlation between EUL and EUD (R^2^ = 0.79 and 0.69 for EUD_b_ and EUD_r_, respectively). In a sample of 20 patients, the average bladder V40 and V50 derived from the EE plans were significantly lower (V40: 30.00 ± 5.76, V50: 14.36 ± 4.00) than the V40 and V50 values derived from manual plans (V40: 36.03 ± 8.02, V50: 19.02 ± 5.42). Compared with the PAP plans, the EE plans produced significantly lower average V30 and Dmean values for the bladder (V30: 50.55 ± 6.33, Dmean: 31.48 ± 1.97 Gy).

**Conclusions:**

Our EEKB prediction models predicted reasonable initial EUD values for the rectum and bladder based on patient-specific geometric EUL values, thereby improving optimization and planning efficiency.

**Supplementary Information:**

The online version contains supplementary material available at 10.1186/s13014-022-02120-4.

## Background

Cervical cancer is the fourth most common cancer in women and results in over 300,000 deaths worldwide [1, 2]. Advances in radiotherapy technology, such as intensity-modulated radiotherapy (IMRT), have reduced treatment-related toxicity for women with locally advanced tumors. To take full advantage of IMRT, high-quality treatment plans are required. In conventional inverse treatment planning, optimization is achieved through a manual trial and error process [3–5]. This conventional planning process is not only very time-consuming but also dependent on the dosimetrist’s skill and experience, thereby subjecting clinical goals to a substantial amount of variation and inconsistency between dosimetrists and institutions [4, 6–9].

To overcome these limitations and improve planning efficiency, several semi-auto- or auto-planning strategies have been proposed in recent years [10–13]. The knowledge-based planning (KBP) approach is one such solution [14–17]. This approach uses previous patients’ plans to create organ-at-risk (OAR) prediction models that can be used to improve OAR sparing for new patients. It has been successfully implemented using commercial software programs such as RapidPlan (Varian Medical Systems, Palo Alto, CA). Tol et al. [14] evaluated the performance of RapidPlan in creating plans for head and neck cancer using volumetric modulated arc therapy (VMAT). They demonstrated that the RapidPlan KBP model generated clinically acceptable treatment plans for patients whose geometric anatomy was within the range of the patients in the model library [14]. Furthermore, Chang et al. [15] demonstrated that the RapidPlan KBP models can improve planning efficiency and create quality IMRT plans for nasopharyngeal cancer patients. The reliable performance of KBP models for breast, rectal, cervical and esophageal cancer auto-planning has also been demonstrated [18–22].

Pinnacle auto-planning (PAP) is another common replacement for conventional manual optimization processes. This auto-planning approach is based on a template optimization tool that mimics the iterative optimization process of an experienced dosimetrist. The efficiency of this auto-planning approach has been demonstrated [17, 23]. Studies have evaluated auto-VMAT treatment planning and IMRT plans for head and neck cancers [17, 23]. However, current PAP approaches have three limitations that need to be overcome. First, in the first step of PAP, dosimetrists must use their experiences and skills to set all of the initialization parameters for both planning target volume (PTV) and OAR. Second, to generate clinically acceptable plans, it is necessary to create additional assistant structures (ASs) to guide the optimization process [23]. Last, but not least, an optimized model generated by PAP cannot take advantage of any existing knowledge that could be used to set the initial optimized parameter values or to optimize them. Therefore, the efficiency of the PAP process and the consistently high-quality of auto-plans cannot be guaranteed.

On the other hand, the conventional iterative optimization process, sets physical dose-volume constraints for all organs. As a variation, Niemierko et al. [24] proposed using an equivalent uniform dose (EUD) as an optimization parameter; this requires the conversion of a nonuniform partial dose distribution to an EUD distribution that can achieve the same cancer-killing effect. An extension, gEUD, calculates doses for normal tissues [25]. Compared with approaches that use conventional dose-volume constraints to optimize plans, using EUD to optimize objects can balance conflict-optimized constraints and achieve an optimal solution based on the optimization system for prostate cancer [26]. In addition, research has demonstrated that a better dose distribution could be produced by combining physical dose-volume constraints with EUD in the IMRT optimization procedure [25, 27]. However, the optimized constraints under EUD generally need to be set manually, although it is possible to use the RapidPlan system to predict EUD values from pre-dose-volume-histogram-knowledge (pre-DVH-knowledge). The performance and efficiency of this method have not been evaluated.

In this study, to overcome the limitation of manually setting initial optimization parameters and to maximize the advantages of using EUD in the optimization process, we developed a novel concept, the equivalent uniform length (EUL), which describes the relationship between the EUD and the geometric anatomy around a tumor target. By correlating EUL and EUD, we established two EUD-EUL-knowledge-based (EEKB) prediction models for the bladder and rectum; one can predict initial optimization parameters and the other can predict EUD values. Furthermore, we tested and evaluated the feasibility and efficiency of a proposed ideal semi-auto-planning workflow that uses these two EE-knowledge-based models to replace conventional initialized dose optimized parameters with predicted initial EUD parameters for the bladder and rectum in the treatment planning of cervical cancer.

## Methods

### Patient selection

Sixty patients with cervical cancer who were treated with IMRT at our institution in 2017 were enrolled in this study of EEKB prediction models. These enrolled patients with high-quality IMRT plans were selected by experienced dosimetrists and physicists purposely for guaranteeing the predicted accurate EUD values of models.

### Planning methods

Patient simulation, contouring techniques, and prescription doses were consistent across all of the patients enrolled in this study. The patients were immobilized in the prone position using a belly board and underwent a simulation computer tomography (CT) scan (Philips Brilliance Big Bore CT, 3 mm slice thickness, 120 kVp, 200 mA, 60 cm field of view). The clinical target volume (CTV) of each patient was manually contoured by an experienced physician, including the primary tumor area, uterus, and the pelvic and para-aortic lymph nodes. The corresponding PTV was created by expanding 0.5 cm symmetrically around the CTV. The OARs, including the bladder, rectum, and femoral heads, were manually contoured (Pinnacle version 9.10, Philips Radiation Oncology Systems, Milpitas, CA).

All of the patients received IMRT at a dose of 59.4 Gy in 33 fractions. All of the original clinically approved IMRT plans were created using seven evenly distributed coplanar fields (each one was 51 degrees) using the direct machine parameter optimization (DMPO) algorithm in the Pinnacle planning system. In addition, all of the plans were normalized to the mean dose of the PTV (the 95% isodose line was set to 59.4 Gy). The highest priorities were given to the protocol criteria for the bladder and rectum that could be achieved without compromising PTV coverage (at least 95% of the PTV must receive 59.4 Gy). The IMRT protocol criteria for the OARs were as follows: bladder V40 Gy (receive 40 Gy or more) < 40% and mean dose *Dmean* < 35 Gy; rectum V40 Gy < 40% and mean dose *Dmean* < 35 Gy; and femoral-head V40 Gy < 5% and mean dose *Dmean* < 25 Gy. All of the original clinically approved IMRT plans were modified manually by experienced dosimetrist after primary plan optimization based on PAP module.

All of the dose calculations were performed with the adaptive convolve algorithm with a calculation grid of 4 mm.

### Developing EUL–EUD knowledge-based prediction models

Niemierko et al. [24] defined EUD using the following equation:

1 $$EUD= {(\frac{1}{N}\sum_{i=1}^{N}{D}_{i}^{a})}^\frac{1}{a}$$

where $N$ is the total pixel number of specific organs; ${D}_{i}$ is the dose received by the *i*th pixel of a specific organ; and $a$ is a specific parameter for describing the dose-volume effect of the tumor or normal tissue. In Eq. (1), when $a=1$, EUD represents the mean dose value. A larger $a$ value is associated with a greater proportion of high dose in the EUD. When $a$ is close to positive infinity or negative infinity, the EUD can be regarded as the approximate maximum dose value or minimum dose value, respectively.

However, based on the characteristic and principle of the overlap volume histogram (OVH), we proposed a novel concept, EUL, as a type of pre-knowledge that can be used to establish an EE-knowledge-based prediction model:

2 $$EUL={(\frac{1}{N}\sum_{i=1}^{N}{L}_{i}^{a})}^\frac{1}{a}$$

where $N$ is the total pixel number of specific organs and ${L}_{i}$ is the shortest length from the PTV to the *i*th pixel of a specific organ. In other words, EUL could be seen as the shortest PTV expansion length that includes the *i*th pixel. $a$ is the same as in the normal tissue-specific parameter $a$ in Eq. (1).

We established two EE knowledge-based prediction models based on EULs (where $a=1$) for the bladder (${EUL}_{b}^{a=1}$) and rectum (${EUL}_{r}^{a=1}$), which we used to predict EUD values (where $a=1$) for these two organs (${EUD}_{b}^{a=1}$, ${EUD}_{r}^{a=1}$). The methodology for generating EE knowledge-based prediction models assumed a direct relationship between the EUL of a specific OAR and the corresponding EUD. The Pinnacle treatment planning system was used to calculate and extract the ${EUL}_{b}^{a=1}$, ${EUL}_{r}^{a=1}$, ${EUD}_{b}^{a=1}$, and ${EUD}_{r}^{a=1}$ for 60 patients using a software program developed in-house with Pinnacle scripts. The EE knowledge-based prediction models for the bladder and rectum were generated using linear regression and Pearson correlation tests that examined the relationships between ${EUL}_{b}^{a=1}$ and ${EUD}_{b}^{a=1}$, and between ${EUL}_{r}^{a=1}$ and ${EUD}_{r}^{a=1}$.

### Evaluating the efficiency of the EE-knowledge-based prediction models and proposed workflow

**Fig. 1:**
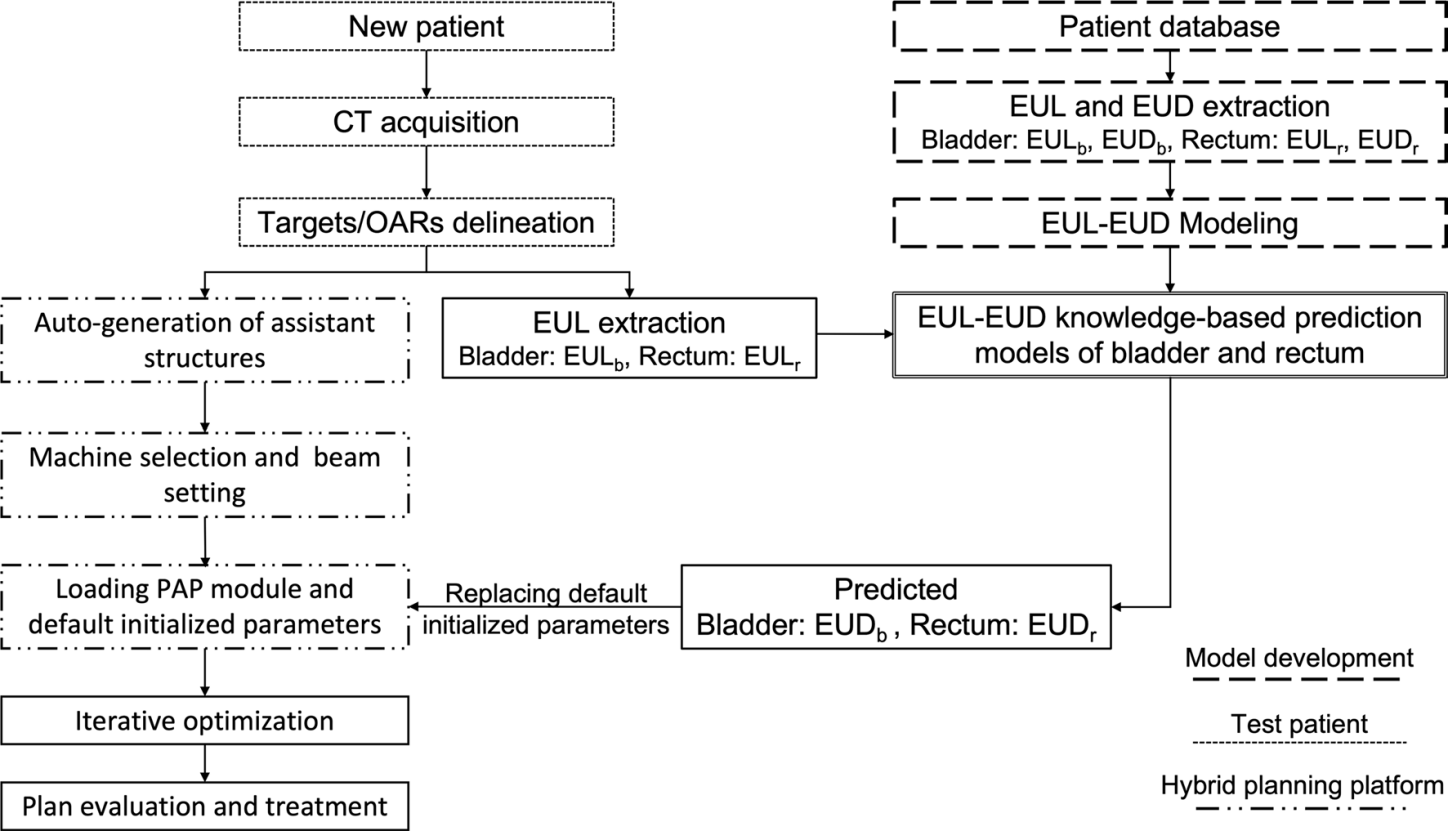
Workflow of EEKB semi-auto-planning

Based on our two EEKB prediction models, we proposed an ideal auto-planning workflow that could efficiently generate clinically acceptable IMRT plans with high consistency (Fig. 1). This auto-planning workflow starts after CT acquisition and targets/OARs delineation. To evaluate the feasibility and efficiency of the two prediction models and the proposed workflow, we used a sample of 20 randomly selected patients with cervical cancer undergoing IMRT at our institution as test patients to develop EEKB prediction models. These test patients were treated at our institution before 2017. Thus, their IMRT plans were designed manually based on Pinnacle Version 9.2 by experienced dosimetrists, no PAP primary optimization was included. The PTV and OARs of these test patients were contoured based on CT scans with criteria identical to those used in clinical practice and as described previous planning section.

**Fig. 2:**
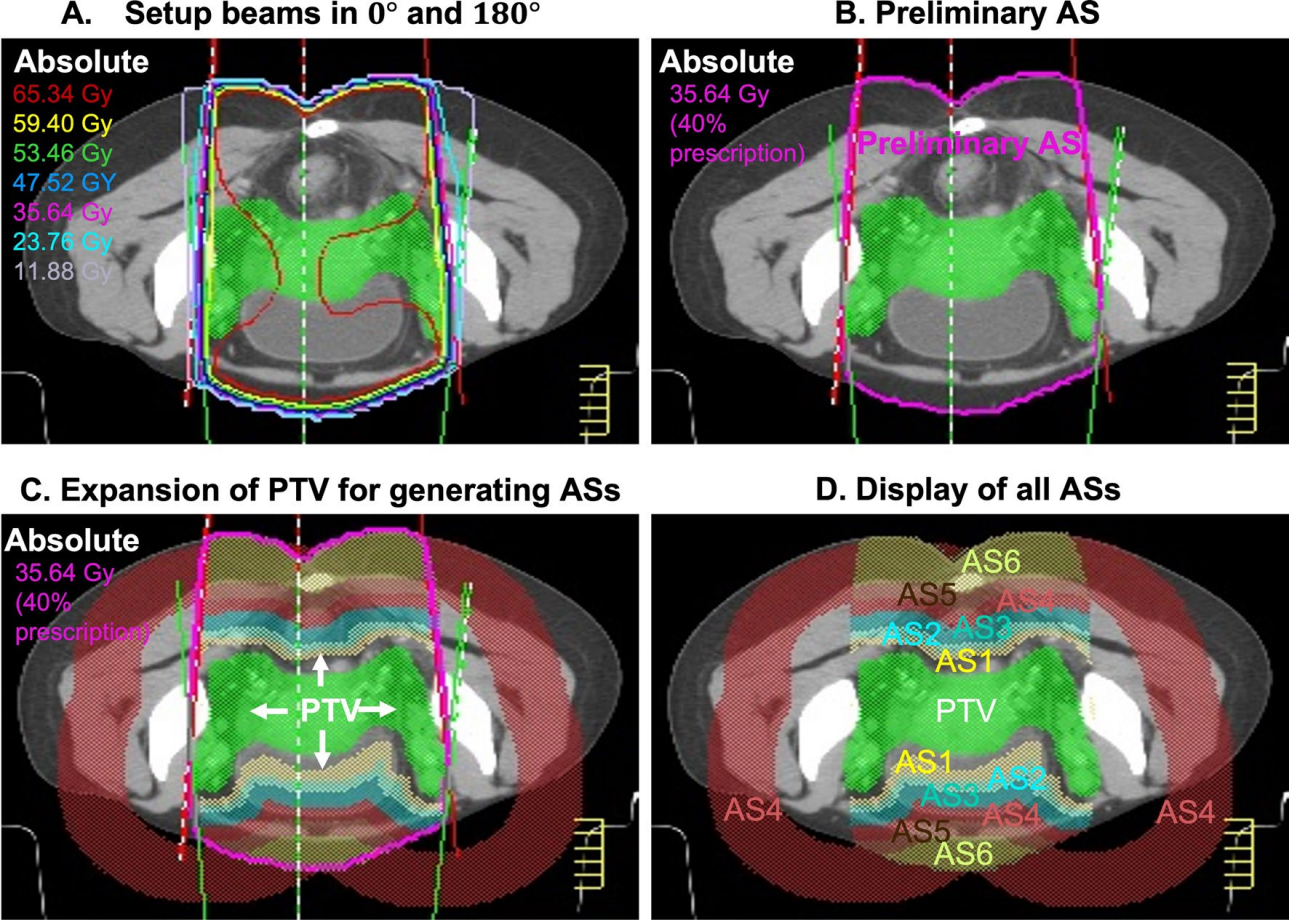
Generating assistance structures. **A** Setup beams at $0^\circ$ and $180^\circ$. **B** A preliminary AS was created at 40% of the prescription dose. **C** AS1 to AS6 were generated by expanding the PTV in incremental steps. **D** All of the ASs

We used the proposed workflow (Fig. 1) to generate EUL-EUD IMRT plans (EE plans) for all 20 test patients. As shown in Fig. 1, after the delineation of the targets and OARs, we created an in-house hybrid planning platform based on a Python software program with Pinnacle scripts. This hybrid planning platform was compatible with the PAP module and Pinnacle treatment planning system. The first step was to automatically generate six assistant structures (AS1–AS6). These ASs controlled the homogeneity of dose distribution around the PTV. Figure 2 shows the AS generation process. First, two beams, at $0^\circ$ and $180^\circ$, were set to adapt the PTV shape to receive the prescription dose (Fig. 2A). Second, based on the distribution line of 40% of the prescription dose, one preliminary AS (pink line Fig. 2B) was created. Then, two ring organs were created by expanding the PTV from 0.5 to 1.5 cm in steps of 0.5 cm. AS1 and AS2 were generated by extracting the overlap between the preliminary AS and the two ring organs. The third, fourth, and fifth ring organs were created by expanding the PTV from 1.5 to 4.5 cm in steps of 1 cm; AS3, AS4, and AS5 were defined as the overlap between the preliminary AS and the ring organs. In addition to the overlap region, AS4 included an additional part: the ring organ generated by expanding the PTV from 3.5 to 4.5 cm, excluding the part that overlapped with the preliminary AS. Finally, AS6 was the preliminary AS after excluding AS1, AS2, AS3, AS4, AS5, and the PTV 0.5 cm expansion. Figure 2C, D illustrate the procedure and all of the ASs.

After the six ASs were generated, the machine selection and beam setting were modularized and added to the platform to simplify the planning procedure. This step provides the option of setting 7 or 9 evenly spaces beams. Together, they represent all of the available treatment machines in our institute.

The last step of this hybrid auto-planning platform was to optimize the plan using the PAP module and corresponding default initial parameters. The initial parameters and planning objectives of the PAP module were as follows: direct machine parameter optimization, 59.4 Gy in 33 fractions as the prescription dose, bladder mean dose 35 Gy (weight high), rectum mean dose 35 Gy (weight high), femoral heads mean dose 20 Gy (weight medium), AS1 max. dose 47 Gy (weight medium), AS2 max. dose 45 Gy (weight medium), AS3 max. dose 40 Gy (weight medium), AS4 max. dose 30 Gy (weight medium), AS4 max. dose 20 Gy (weight medium), and AS5 max. dose 10 Gy (weight high). The initial values of ASs optimized objects were proposed and set based on our clinical experience.

We used the extracted ${EUL}_{b}^{a=1}$ and ${EUL}_{r}^{a=1}$ of these test patients as inputs for the EEKB prediction models that were used to estimate the predicted ${EUD}_{b}^{a=1}$ and ${EUD}_{r}^{a=1}$. Then, the conventional initial dose optimization parameter of the bladder and rectum identified by the PAP module were replaced with ${EUD}_{b}^{a=1}$ and ${EUD}_{r}^{a=1}$, respectively. Thus, the optimization procedure incorporated the predicted initial EUD parameters for the rectum and bladder and the conventional PAP initial dose parameters for the other organs. The optimization process stopped when it reached the maximum iterations within PAP. No extra manual modifications or improvements were made to the EE-plans after the PAP optimization.

We also generated PAP plans (with an optimization process based on the PAP default initial parameters) for all of the test patients. The optimization process was the same as for the EE plans but was based on PAP default initial parameters, which were the conventional dose parameters. In addition, all of the test patients’ clinically approved IMRT plans (manually designed by different dosimetrists) were included in our evaluation.

By comparing the consistency and quality of the manual, PAP, and EE plans, we evaluated the efficiency and feasibility of the two proposed prediction models, the hybrid auto-planning platform, and the proposed workflow (Fig. 1). Plan consistency was calculated using a linear regression of all of the tested patients’ plans. Plan quality was evaluated using dose-volume criteria.

### Statistical analysis

The relationships between ${EUL}_{r}^{a=1}$ and ${EUD}_{r}^{a=1}$, and between ${EUL}_{b}^{a=1}$ and ${EUD}_{b}^{a=1}$ were measured using the Pearson correlation test and a linear regression, respectively. A paired t-test was used to validate plan quality, and a *p*-value of *p* < 0.05 was considered statistically significant. All of the statistical tests were two-tailed and performed using the Origin software (OriginLab Corporation, Northampton, US).

## Results

**Fig. 3:**
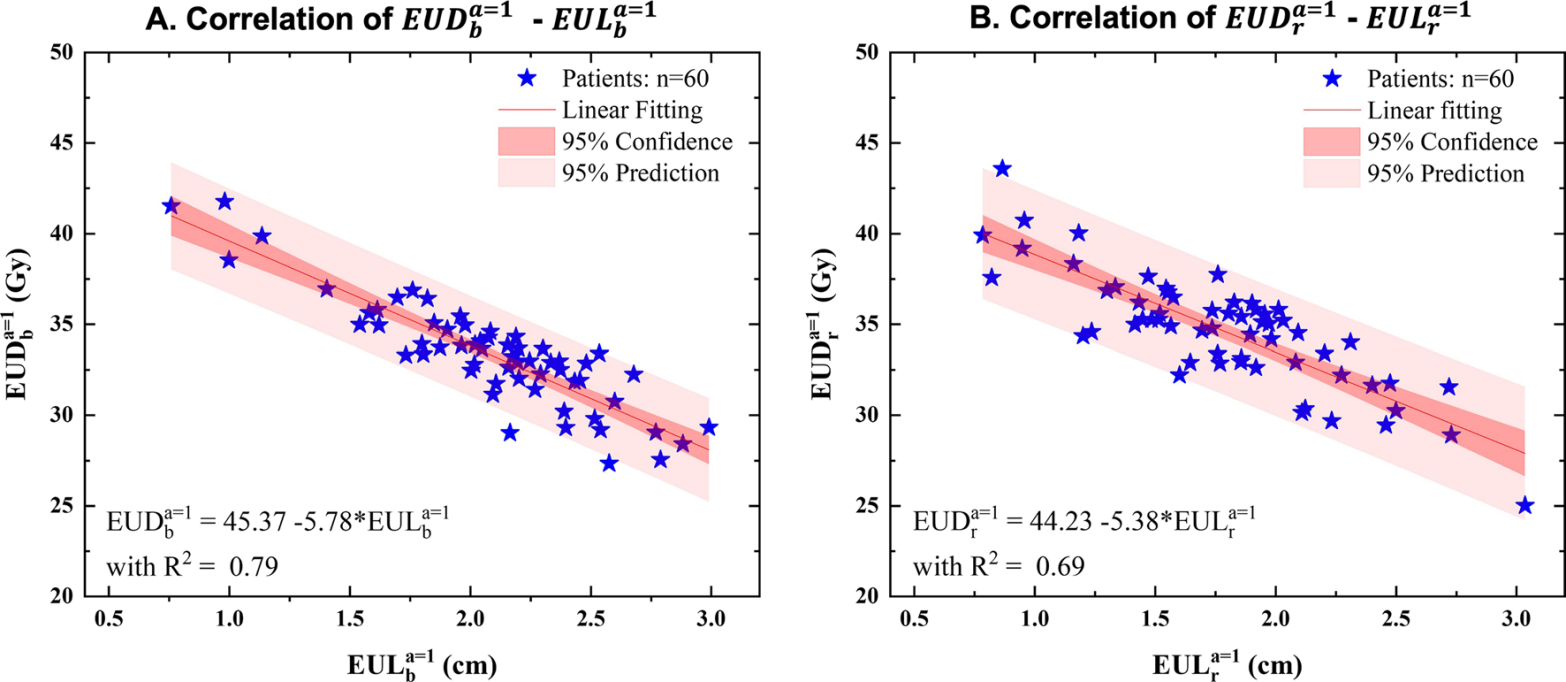
EUL–EUD knowledge-based prediction models for rectum and bladder. The EUL variables (${EUD}_{b}^{a=1}$ and ${EUD}_{r}^{a=1}$) and the EUD variables (${EUL}_{b}^{a=1}$ and ${EUL}_{r}^{a=1}$) of 60 patients were extracted. Linear regression was used to analyze the correlation between the variables with a 95% prediction range (light red shading) and 95% confidence interval (dark red shading). **A** Showed the EEKB of bladder with $a=1$. **B** Showed the EEKB of rectum with $a=1$

Figure 3 shows the correlations between the EUL variables (${EUD}_{b}^{a=1}$, ${EUD}_{r}^{a=1}$) and the corresponding EUL variables (${EUL}_{b}^{a=1}$, ${EUL}_{r}^{a=1}$) determined by the linear regression. Each star represents the EUD and EUL variables of one patient. These correlations were determined as follows:

3 $${EUD}_{b}^{a=1}=45.37-5.78*{EUL}_{b}^{a=1} \left({R}^{2}=0.79\right)$$

4 $${EUD}_{r}^{a=1}=44.23-5.38* {EUL}_{r}^{a=1} \left({R}^{2}=0.69\right).$$

${EUD}_{b}^{a=1}$ and ${EUD}_{r}^{a=1}$ were calculated by setting $a=1$ in the models. These two functions, as defined in Eqs. (3) and (4), were the predicting lines for the ${EUD}_{b}^{a=1}$–${EUL}_{b}^{a=1}$ and ${EUD}_{r}^{a=1}$–${EUL}_{r}^{a=1}$ knowledge based prediction models, respectively.

**Fig. 4:**
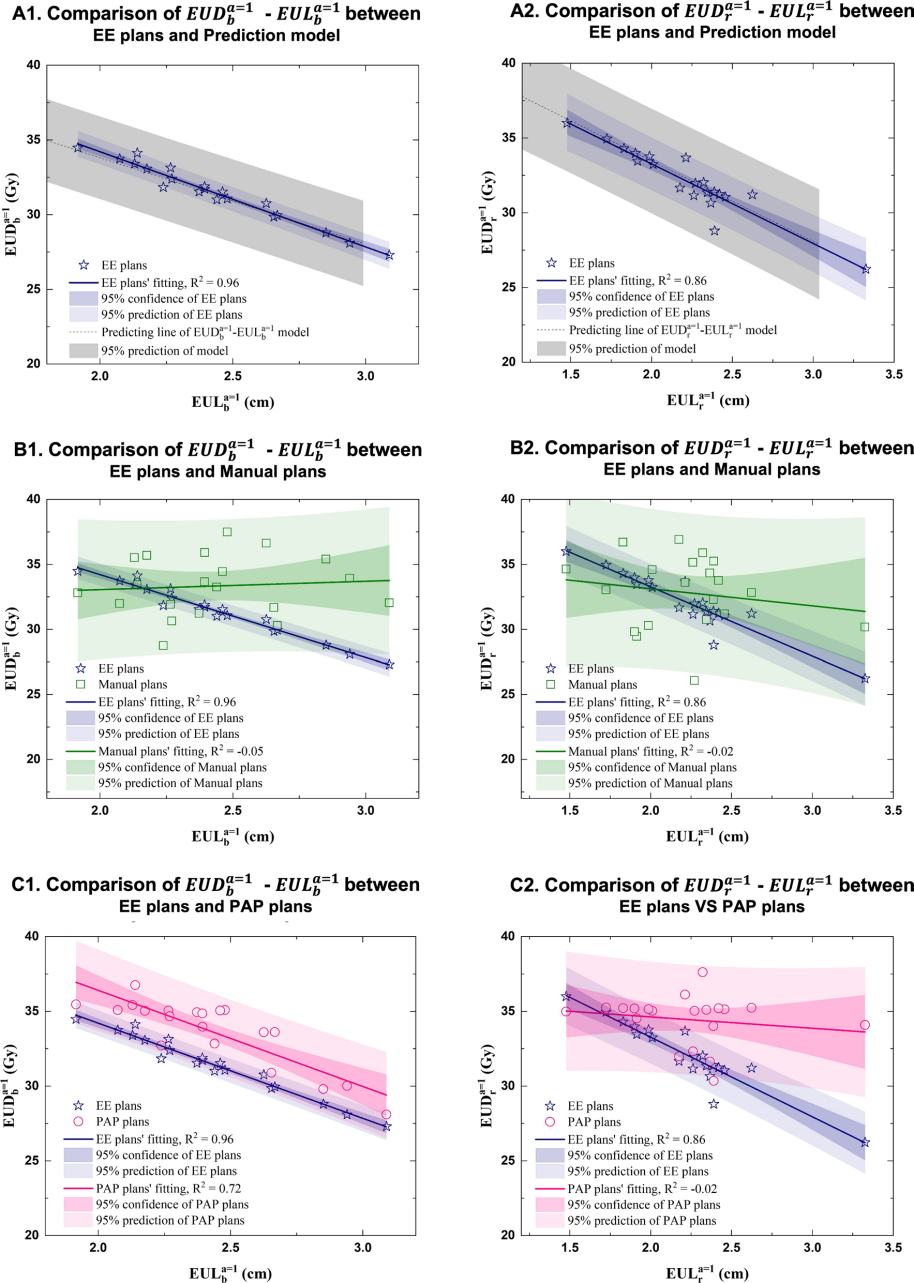
The blue lines, light blue shading, and dark blue shading in **A1**, **A2**, **B1**, **B2**, **C1**, and **C2** indicate the linear fitting lines, 95% prediction range, and 95% confidence intervals of the ${EUD}_{b}^{a=1}-{EUL}_{b}^{a=1}$ and ${EUD}_{r}^{a=1}-{EUL}_{r}^{a=1}$ of EE plans, respectively. The green lines, light green shading, and dark green shading in **B1** and **B2** indicate the linear fitting lines, 95% prediction range, and 95% confidence intervals of the ${EUD}_{b}^{a=1}-{EUL}_{b}^{a=1}$ and ${EUD}_{r}^{a=1}-{EUL}_{r}^{a=1}$ of manual plans, respectively. The pink lines, light pink shading, and dark pink shading in **C1** and **C2** indicate the linear fitting lines, 95% prediction range, and 95% confidence intervals of the ${EUD}_{b}^{a=1}-{EUL}_{b}^{a=1}$ and ${EUD}_{r}^{a=1}-{EUL}_{r}^{a=1}$ of PAP plans, respectively

Plan consistency was evaluated by comparing the ${EUD}_{b}^{a=1}$–${EUL}_{b}^{a=1}$ and ${EUD}_{r}^{a=1}$ –${EUL}_{r}^{a=1}$ values of the EE plans (based on the EEKB prediction model), manual plans, and PAP plans. Figure 4 shows the results of the comparison. The stars, rectangles, and circles in Fig. 4 represent the EUD and EUL variables of the patients’ EE, manual, and PAP plans, respectively. ${EUD}_{b}^{a=1}$ and ${EUD}_{r}^{a=1}$ were calculated by setting $a=1$. The gray shading and dotted lines in Fig. 4A1 and A2 indicate the 95% prediction interval and the predicting line of the ${EUD}_{b}^{a=1}$ –${EUL}_{b}^{a=1}$ and ${EUD}_{r}^{a=1}$ –${EUL}_{r}^{a=1}$ knowledge based prediction models, respectively.

Figure 4A1, B1, and C1 compare the plan consistency of the ${EUD}_{b}^{a=1}$ –${EUL}_{b}^{a=1}$ in the EE, manual, and PAP plans, respectively. Linear regression was used to analyze the correlations and consistency between the variables with a 95% prediction range (light shading) and 95% confidence interval (dark shading). The ${R}^{2}$ of the ${EUD}_{b}^{a=1}$ –${EUL}_{b}^{a=1}$ fitting lines of the EE, manual, and PAP plans were 0.96, -0.05, and 0.72, respectively. Similarly, Fig. 4A2, B2, and C2 compare the ${EUD}_{r}^{a=1}$ –${EUL}_{r}^{a=1}$ values of the EE, manual, and PAP plans, respectively. The ${R}^{2}$ of the ${EUD}_{r}^{a=1}$ –${EUL}_{r}^{a=1}$ fitting lines of the EE, manual, and PAP plans were 0.86, − 0.02, and − 0.02, respectively. The plans based on the EEKB model had higher consistency than the manual or PAP plans, as shown by the high ${R}^{2}$ and good fit of their ${EUD}_{b}^{a=1}$ –${EUL}_{b}^{a=1}$ and ${EUD}_{r}^{a=1}$ –${EUL}_{r}^{a=1}$ values.

As shown in Table 1, EE plans produced a lower average V50 and V40 for the bladder ($mean\pm standard deviation$: $14.36 \pm 4.00$ and$30.00 \pm 5.76$, $p values were<0.01$ and$0.02$, respectively) than the manual plans ($mean\pm standar deviation$: $19.02 \pm 5.42$ and$36.03 \pm 8.02$, $p values were 0.01$ and$0.02$, respectively). Compared to the PAP plans, the ${D}_{mean}$ and V30 in the EE plans were significantly lower ($p<0.01$ for both parameters). Specifically, the ${D}_{mean } mean\pm standard deviation$ for the EE and PAP plans were $31.48\pm 1.97 \mathrm{Gy}$ and$33.65 \pm 2.27 \mathrm{Gy}$, respectively. The $V30 mean\pm standard deviation$ for the EE and PAP plans were $50.55\pm 6.33$ and$57.87 \pm 6.43$, respectively. For the rectum, the EE plans produced lower values for V30, V40, and ${D}_{mean}$ ($mean\pm standard deviation$ of V30, V40, and ${D}_{mean}$ were $50.43\pm 9.44, 28.31 \pm 6.91,$ and$32.11 \pm 2.21$, respectively) than the PAP plans ($mean\pm standard deviation$ of V30, V40, and ${D}_{mean}$ were $58.70\pm 7.62, 33.85\pm 6.06,$ and$34.46 \pm 1.68$, respectively; $p$ values were$<0.01$, 0.03, and$<0.01$, respectively). The mean V40 and V50 produced by the EE plans were also significantly lower ($mean\pm standard deviation$ of V40 and V50 were $28.31\pm 6.91$ and$10.94 \pm 4.69$, respectively) than those produced by the manual plans ($mean \pm standard deviation$ of V40 and V50 were $34.15\pm 7.02$ and$15.75 \pm 5.79$, respectively; $p$ values were $0.02$ and$0.01$, respectively). In addition, although the prediction models only aimed to pre-set the EUD constraints of the bladder and rectum, the ${D}_{mean}$ of the left femoral head was also significantly lower in the EE plans than in the manual plans.

**Table 1:** Comparison of EE, PAP, and manual plans

Structures	Parameters	EE plans	PAP plans	Manual plans	p (EE vs. PAP)	p (EE vs. M)
PTV	V95 (%)	95.38 ± 0.92	96.04 ± 0.73	95.57 ± 1.41	0.13	0.83
	HI	0.11 ± 0.01	0.11 ± 0.01	0.11 ± 0.01	0.10	0.82
	CI	0.74 ± 0.04	0.74 ± 0.04	0.73 ± 0.04	1.00	0.45
Bladder	V20 (%)	72.55 ± 6.58	77.94 ± 8.14	71.55 ± 7.87	0.07	0.91
	V30 (%)	50.55 ± 6.33	57.87 ± 6.43	53.04 ± 8.06	< 0.01*	0.50
	V40 (%)	30.00 ± 5.76	35.10 ± 6.55	36.03 ± 8.02	0.06	0.02*
	V50 (%)	14.36 ± 4.00	16.69 ± 4.51	19.02 ± 5.42	0.27	< 0.01*
	V60 (%)	3.45 ± 1.90	3.96 ± 2.02	4.29 ± 1.92	0.69	0.36
	Dmean (Gy)	31.48 ± 1.97	33.65 ± 2.27	32.84 ± 2.81	< 0.01*	< 0.18
Rectum	V20 (%)	81.77 ± 9.24	88.40 ± 8.68	79.65 ± 9.75	0.07	0.75
	V30 (%)	50.43 ± 9.44	58.70 ± 7.62	54.37 ± 7.78	< 0.01*	0.30
	V40 (%)	28.31 ± 6.91	33.85 ± 6.06	34.15 ± 7.02	0.03*	0.02*
	V50 (%)	10.94 ± 4.69	13.77 ± 5.01	15.75 ± 5.79	0.20	0.01*
	V60 (%)	1.08 ± 0.98	1.54 ± 1.46	1.55 ± 1.27	0.49	0.47
	Dmean (Gy)	32.11 ± 2.21	34.46 ± 1.68	33.34 ± 2.31	< 0.01*	0.16
Femoral head-L	V20 (%)	43.49 ± 4.52	43.92 ± 4.42	47.38 ± 4.52	0.97	0.08
	V30 (%)	15.23 ± 4.25	14.37 ± 3.86	15.51 ± 5.70	0.83	0.98
	V40 (%)	2.28 ± 1.40	2.19 ± 1.71	2.97 ± 2.67	0.99	0.53
	V50 (%)	0.10 ± 0.15	0.11 ± 0.21	0.20 ± 0.43	0.99	0.52
	V60 (%)	0.00 ± 0.00	0.00 ± 0.00	0.00 ± 0.00	–	–
	Dmean (Gy)	20.12 ± 0.64	20.16 ± 0.69	21.08 ± 1.41	0.99	< 0.01*
Femoral head-R	V20 (%)	41.49 ± 9.81	41.75 ± 9.79	45.31 ± 11.97	1.00	0.49
	V30 (%)	14.00 ± 6.12	14.01 ± 6.38	14.12 ± 6.74	1.00	1.00
	V40 (%)	2.42 ± 2.11	2.61 ± 2.41	3.01 ± 3.01	0.97	0.75
	V50 (%)	0.16 ± 0.37	0.14 ± 0.35	0.14 ± 0.28	1.00	0.99
	V60 (%)	0.00 ± 0.00	0.00 ± 0.00	0.00 ± 0.00	–	–
	Dmean (Gy)	19.81 ± 1.84	19.96 ± 1.58	20.72 ± 2.31	0.96	0.30

Mean ($\pm standard deviation$) planning target volume (PTV) coverage in %; conformity index (CI) and homogeneity index (HI), organs at risk (OARs) in % and Gy with *p*-values for the sample test patients. Statistically significant differences are in red with an asterisk

**Fig. 5:**
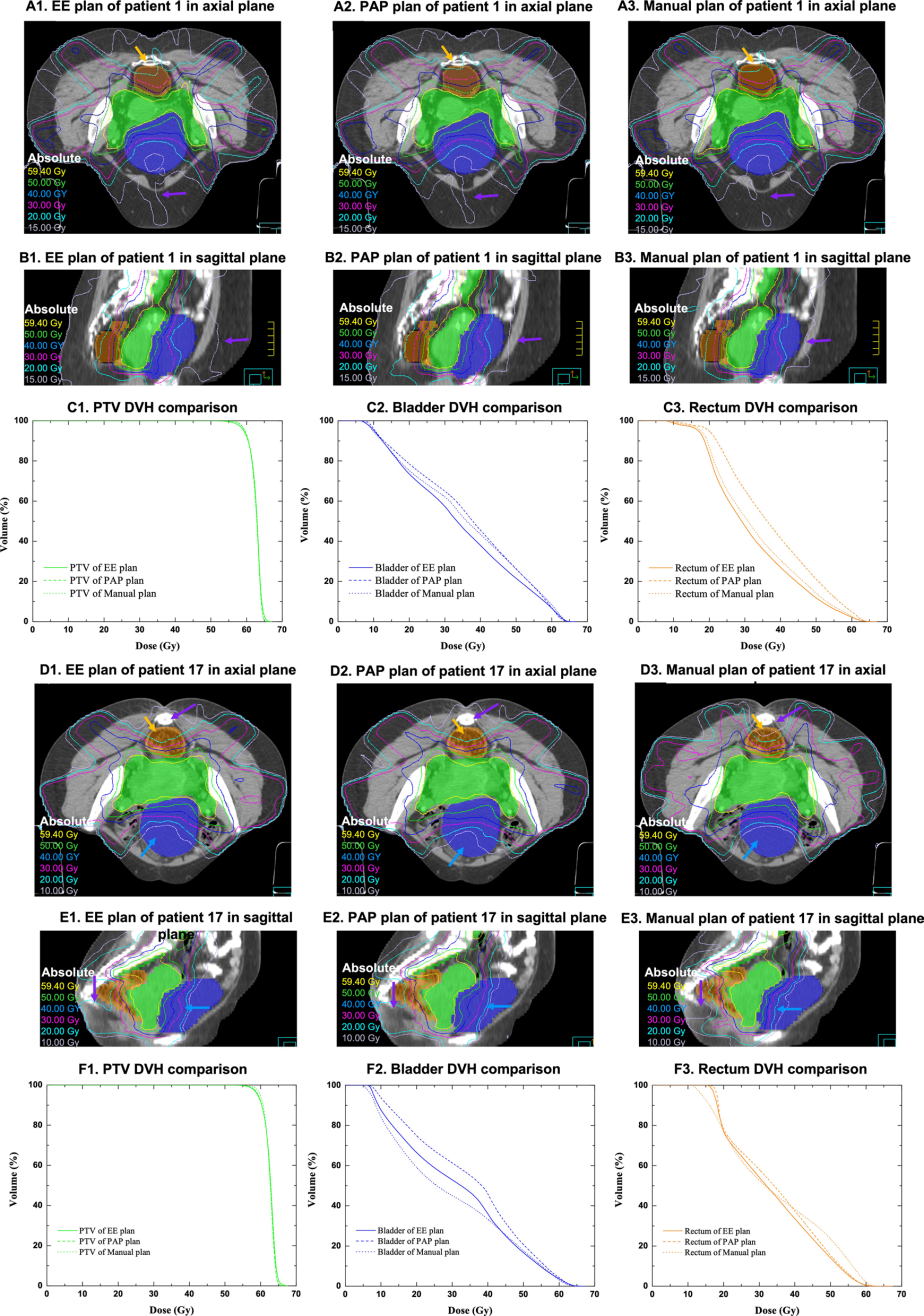
Dose distribution and DVH comparison of patients No.1 and No.17. The orange, green, and blue areas are the rectum, PTV, and bladder, respectively. From **A1** to **B3**, the yellow, green, blue, pink, cyan, and grey isodose lines represent prescription doses of 59.4 Gy, 50.00 Gy, 40.00 Gy, 30.00 Gy, 20.00 Gy, and 15.00 Gy, respectively. The orange arrows in **A1** to **A3** and **D1** to **D3** represent increased doses to the rectum in the low dose region (20.00 Gy). The purple arrows in **A1** to **A3**, **B1** to **B3**, **D1** to **D3**, and **E1** to **E3** indicate decreased doses to the normal tissue in the low dose region (15.00 Gy). In **C1** to **C3**, the solid, dashed, and dotted lines represent the organ’s DVH of the EE, PAP, and manual plans, respectively. In **D1** to **E3**, the yellow, green, blue, pink, cyan, and grey isodose lines represent prescription doses of 59.4 Gy, 50.00 Gy, 40.00 Gy, 30.00 Gy, 20.00 Gy, and 10.00 Gy, respectively. The blue arrows in **D1** to **D3** and **E1** to **E3** represent the different dose distributions in the bladder in the EE, PAP and manual plans. The purple arrows in **D1** to **D3** and **E1** to **E3** indicate the decreased dose to normal tissue in the low dose region (10.00 Gy). In **F1** to **F3**, the solid, dashed, and dotted lines represent the organ’s DVH in the EE, PAP, and manual plans, respectively

Figure 5 compares the results of patients’ No.1 and No. 17. The dose distributions of the EE, PAP and manual plans of patient No. 1 are shown in Fig. 5A1–A3 and B1–B3, and those of patient No. 17 are shown in 5D1–D3 and E1–E3. The orange, green, and blue areas represent the rectum, PTV, and bladder, respectively. The yellow, green, blue, pink, cyan, and grey isodose lines represent prescription doses of 59.4 Gy, 50.00 Gy, 40.00 Gy, 30.00 Gy, 20.00 Gy, and 15.00 Gy, respectively. For test patient No.1, as shown by the orange arrows in Fig. 5A1–A3, there were higher received doses in the rectum in the low dose region (20.76 Gy) in the PAP plan and the manual plan than in the EE plan. However, as shown by the purple arrows in Fig. 5A1–A3 and B1–B3, there was a lower received dose in the normal tissue in the low dose region (15.00 Gy) under the PAP and manual plans than under the EE plan. In Fig. 5C1–C3, the solid, dashed, and doted lines represent the PTV (C1), bladder (C2) or rectum (C3) DVH of the EE, PAP, and manual plans, respectively. As Fig. 5C2 and C3 show, the bladder and rectum received the lowest dose in the EE plan while maintaining a similar PTV coverage.

Similarly, for test patient No. 17, as shown by the orange arrows in Fig. 5D1–D3, the PAP and manual plans had higher received doses in the rectum in the low dose region (20.00 Gy) than the EE plan. In contrast, the blue arrows in Fig. 5D1–D3 and E1–E3 represent the different dose distribution for the bladder in the EE, PAP, and manual plan. The bladder received the lowest dose in the manual plan and the highest dose in the PAP plan. Furthermore, the purple arrows in Fig. 5D1–D3 and E1–E3 show that the 10.00 Gy isodose line covered less normal tissue in the manual plan than in the PAP and EE plans. The DVH of the PTV, bladder, and rectum are shown in Fig. 5F2 and F3. In Fig. 5F1–F3, the solid, dashed, and dotted lines represent the PTV (5F1), bladder (5F2), or rectum (5f3) DVH of the EE, PAP, and manual plans, respectively. In the manual plan, the bladder and rectum received lower doses in the lower dose region than in the EE plan. The rectum received a higher dose in the manual plan than in the EE plan.

## Discussion

We developed a novel concept, EUL, which could link patient-specific geometric anatomy with EUD values. Furthermore, we built EEKB prediction models for the bladder and the rectum by correlating ${EUD}_{b}^{a=1}$ –${EUL}_{b}^{a=1}$ and ${EUD}_{r}^{a=1}$ –${EUL}_{r}^{a=1}$. Instead of adopting traditional dose-volume optimized parameters as initial optimized objects, we developed and evaluated an ideal workflow that used these two prediction models to estimate the initial optimization parameters for the two organs and to efficiently generate clinically acceptable treatment plans for patients with cervical cancer. This study provides valuable insights into the feasibility and dosimetric advantages of this semi-auto-planning workflow for identifying clinical dose constraints, and thereby improving the design efficiency and consistency of treatment plans.

Our EUL concept is based on the same principle as the OVH, which quantifies the geometric relationship between the PTV and surrounding OARs. Previous research has shown that using OVH data from a treated-patient library can predict the possible DVH constraints for a new patient [28]. This is the fundamental principle used in constructing OVH prediction models by correlating OVH and DVH; those models can be used for automated treatment planning procedures [29–32]. However, these automated treatment planning procedures are based on dose-volume optimized objects. We expanded the application of these principles by building a prediction model for setting the initial optimized objects, which we call EUD. Our results show that EUL is a more efficient metric than OVH for building the link between EUD values and complicated geometric anatomy around the PTV target. By correlating the EUL and EUD of the bladder and rectum, our results suggest that EUL has promise for predicting relevant, patient-specific initial EUD values for these two organs, which could be used as optimized objects in plan optimization. Compared with manually setting the initial EUD value according to dosimetrists’ experience, the predicted EUD values output by our EE prediction models are personally customized and optimized based on pre-knowledge of each patient’s tumor and surrounding normal tissue anatomy. The results also imply that setting EUD values as optimized objects for some OARs and setting dose-volume constraints for the rest of the organs could be another planning strategy that generates competitive treatment plans. In general, our EE plans are better in sparing the radiation dose of the rectum (V40, V50) and bladder (V40, V50) than typical manual plans. However, in specific patient cases, such as patient No. 17, the rectum and bladder received higher doses in the low region in the EE plans than in the manual plan, because the two EEKB prediction models assume the EUD represents the mean dose of the OARs. During the optimization process, the predicted EUD values as optimized objects were designed to lower the whole DVH curve, not to optimize or control a local section of the dose region.

After developing and testing these two EEKB prediction models, we built a hybrid platform and proposed a workflow to predict patient-specific initial ${EUD}_{b}^{a=1}$ and ${EUD}_{r}^{a=1}$ values based on corresponding EULs. This is one more tool that can be applied in radiation oncology to improve precision and further customize the setting of the initial optimized objects value. Our proposed workflow also addresses the urgent need for auto-planning in clinical practice that draws on accumulated clinical plan data, as the initial set of optimized objects depends on dosimetrists’ experience. In addition, our hybrid planning platform is based on Pinnacle scripts and Python codes that are compatible and available in the Solaris operating system. It would be possible to expand the application of this workflow to any institutions that have Pinnacle3 workstations. Similarly, this flexible hybrid platform could be easily extended and modified for application in different cases such as the treatment of head-and-neck cancers or other planning techniques such as VMAT.

There are two considerable factors contribute to the major difference between EE plans and manual plans. First, the manual plans of tested patients were generated manually without any PAP optimization. However, the patients’ plans enrolled for building EEKB models were designed by incorporating PAP module. Therefore, the manual plans of tested patients totally depended on dosimetrist experiments, whereas the EE plans accounts for EEKB models. Secondly, in our proposed workflow, the application of ASs and setting corresponding optimized parameters aimed to control the dose fall-off pattern out of PTV and limit the dose received by bladder and rectum in anterior and posterior direction. For these two purposes, we defined the ASs and initialized the optimized parameters according to our clinical experiments. Therefore, compared with manual plans, the application of these ASs in optimized process contributed to sparing dose, thereby generating a better plan, such as EE plans.

Furthermore, even though the same ASs definition and corresponding optimized parameters setting were used in the optimization procedure of PAP plans, EE plans still keep better plans quality and consistency compared with PAP plans. Thus, the better plans quality and consistency of EE plans are not only because of the application of ASs in optimization but also comes of the accurate prediction of EEKB models.

This study has several limitations. First of all, as all 60 patients involved in the model database were treated at our institution using the same criteria for PTV delineation, plan evaluation, etc. There may be bias in the radiation treatment methods. Therefore, the two EEKB prediction models derived from these training data should be considered conservative. In addition, in the optimization process, only the rectum and bladder were optimized using the predicted initial EUD value, as we only built the EEKB prediction models for these two organs with $a=1$. Literally, this precited EUD value is equal to the mean dose value. Followed the same concepts, we build another EEKB models of rectum and bladder with $a=0.5$ and $a=2$, respectively (shown in supplementary files). It turned out that EUD and EUL kept a well linear relationship with different $a$ values. In other words, it is feasible to use linear regression for correlating EUD and EUL with different $a$ values, thereby predicting EUD values based on corresponding EUL as we proposed. But there are two reasons for why we did not test and show the accuracy and performance of these EEKB models ($a=0.5$ and $a=2$). First one is the packaged software, Pinnacle, prohibited the users added customized optimized objects, such as gEUD, to PAP module. Thus, the feasibility of our proposed workflow and strategy is tested and evaluated with EUD only when $a=1$. This is the main limitation of the present study. Plus, the EEKB prediction models were established with different $a$ values ($a=0.5$ and $a=2$) by using same 60 patients whose treatment plans were optimized by using mean dose (${EUD}^{a=1}$) as optimized objects in PAP modules. Thus, the ${R}^{2}$ of these EEKB predicted modules ($a=0.5$ and $a=2$) were reduced compared with the ${R}^{2}$ of the EEKB predicted modules with $a=1$. It is proved that, for maintaining high predicted accuracy and well linear correlation, the establishment of EEKB predicted modules with different $a$ values are required IMRT plans optimized with objects in same $a$ value as well. At last, the rest of the OARs were set and optimized using conventional dose-volume optimized objects. Therefore, further improvement and modification of this semi-auto-planning workflow are necessary to build more prediction models for all OARs with different values. This would make it possible to describe different biological dose-volume characteristics. This will ultimately result in more efficient estimations of initial EUD values and better-quality plans.

## Conclusions

In this study, we proposed a novel concept, EUL, for describing geometric anatomy. We built two EEKB prediction models that predict the initial EUD values of the rectum and bladder as optimized objects for designing clinically acceptable treatment plans for patients with cervical cancer. Our results provide valuable insights into the dosimetric advantages of our proposed semi-auto-planning workflow, which may improve the plans’ consistency and planning efficiency.

## Supplementary Information

**Additional file 1: Fig. 6.** EUL–EUD knowledge-based prediction models with different “a” values for rectum and bladder. The EUL variables (${EUL}_{b}^{a=0.5}, {EUL}_{b}^{a=2}, {EUL}_{r}^{a=0.5}, {EUL}_{r}^{a=2}$) and the EUD variables (${EUD}_{b}^{a=0.5}, {EUD}_{b}^{a=2}, {EUD}_{r}^{a=0.5}, {EUD}_{r}^{a=2}$) of 60 patients were extracted. Linear regression was used to analyze the correlation between the variables with a 95% prediction range (light red shading) and 95% confidence interval (dark red shading). **A** Showed the EEKB of bladder with *a*=0.5. **B** Showed the EEKB of rectum with *a*=0.5. **C** Showed the EEKB of bladder with *a*=2. **D** Showed the EEKB of rectum with *a*=2.

## Data Availability

The datasets used and analyzed during the current study are available from the corresponding author on reasonable request.

## Abbreviations

IMRT: Intensity-modulated radiotherapy
KBP: Knowledge-based planning
OAR: Organ-at-risk
VMAT: Volumetric modulated arc therapy
PAP: Pinnacle auto-planning
PTV: Planning target volume
ASs: Assistant structures
EUD: Equivalent uniform dose
EUL: Equivalent uniform length
EEKB: EUD-EUL-knowledge-based
CT: Computer tomography
CTV: Clinical target volume
DMPO: Direct machine parameter optimization
OVH: Overlap volume histogram
