# Supplementary material for: A novel knowledge-based prediction model for estimating an initial equivalent uniform dose in semi-auto-planning for cervical cancer
Source: Radiat Oncol. 2022 Aug 29;17:151. doi: 10.1186/s13014-022-02120-4 (PMC9426003; doi:10.1186/s13014-022-02120-4)
Supplement: Supplementary file 1 — Additional file 1: Fig. 6. EUL–EUD knowledge-based prediction models with different “a” values for rectum and bladder. The EUL variables (${EUL}_{b}^{a=0.5}, {EUL}_{b}^{a=2}, {EUL}_{r}^{a=0.5}, {EUL}_{r}^{a=2}$EULba=0.5,EULba=2,EULra=0.5,EULra=2) and the EUD variables (${EUD}_{b}^{a=0.5}, {EUD}_{b}^{a=2}, {EUD}_{r}^{a=0.5}, {EUD}_{r}^{a=2}$EUDba=0.5,EUDba=2,EUDra=0.5,EUDra=2) of 60 patients were extracted. Linear regression was used to analyze the correlation between the variables with a 95% prediction range (light red shading) and 95% confidence interval (dark red shading). A Showed the EEKB of bladder with a=0.5. B Showed the EEKB of rectum with a=0.5. C Showed the EEKB of bladder with a=2. D Showed the EEKB of rectum with a=2. [file 13014_2022_2120_MOESM1_ESM.docx]

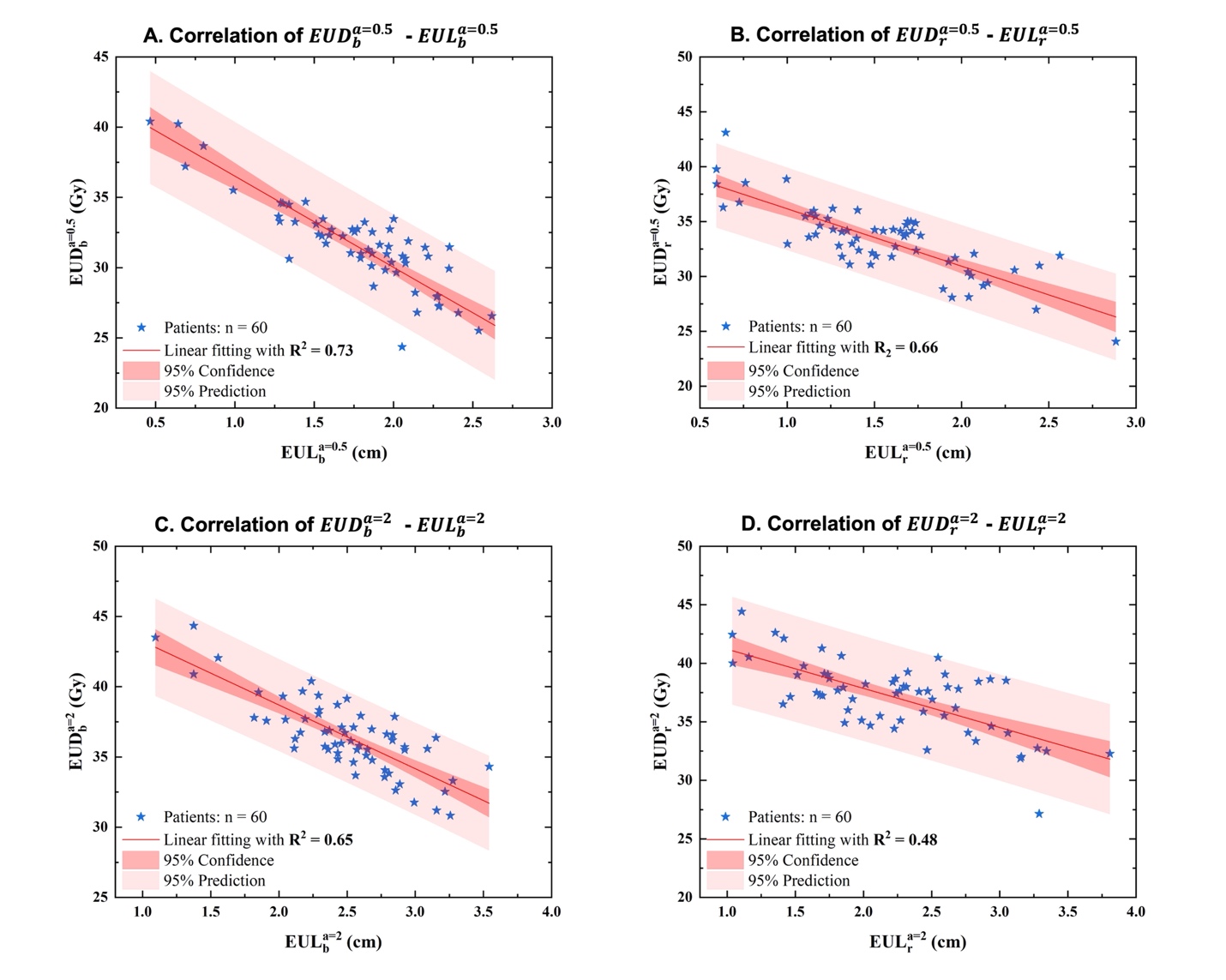


Figure 6. The comparison of EUL-EUD knowledge-based prediction models with different $a$ values. The EUL variables (${EUD}_{b}^{a=0.5}$ and ${EUD}_{r}^{a=0.5}$_,_ ${EUD}_{b}^{a=2}$ and ${EUD}_{r}^{a=2}$) and the EUD variables (${EUL}_{b}^{a=0.5}$ and ${EUL}_{r}^{a=0.5}$_,_ ${EUL}_{b}^{a=2}$ and ${EUL}_{r}^{a=2}$) of 60 patients were extracted. Linear regression was used to analyze the correlation between the variables with a 95% prediction range (light red shading) and 95% confidence interval (dark red shading). A) and B) showed the correlation between ${EUD}_{b}^{a=0.5}$ and ${EUL}_{b}^{a=0.5}$ _,_ ${EUD}_{r}^{a=0.5}$ and ${EUL}_{r}^{a=0.5}$_,_ respectively. Similarly, C) and D) showed the correlation between ${EUD}_{b}^{a=2}$ and ${EUL}_{b}^{a=2}$, ${EUD}_{r}^{a=2}$ and ${EUL}_{r}^{a=2}$_,_ respectively.

The correlations were determined as follows:

${EUD}_{b}^{a=0.5}=42.99-6.48*{EUL}_{b}^{a=0.5} \left( R^{2}=0.73 \right)$ (5)

${EUD}_{r}^{a=0.5}=41.39-5.23* {EUL}_{r}^{a=0.5} \left( R^{2}=0.66 \right)$. (6)

${EUD}_{b}^{a=2}=47.77-4.53*{EUL}_{b}^{a=2} \left( R^{2}=0.65 \right)$ (7)

${EUD}_{r}^{a=2}=44.54-3.34* {EUL}_{r}^{a=2} \left( R^{2}=0.48 \right)$. (8)

These four functions, as defined in Equations (5) to (8) could be considered as the predicting lines for the ${EUD}_{b}^{a=0.5}$ –${EUL}_{b}^{a=0.5}$, ${EUD}_{r}^{a=0.5}$ –${EUL}_{r}^{a=0.5}$, ${EUD}_{b}^{a=2}$ –${EUL}_{b}^{a=2}$ and ${EUD}_{r}^{a=2}$ –${EUL}_{r}^{a=2}$ knowledge based prediction models, respectively.

These EEKB predicted modules showed that the EUD and EUL kept a well linear relationship with different $a$ values. Although not as good as ${EUD}^{a=1}$ - ${EUL}^{a=1}$ , but it is well fitting. This proved that EUD with different $a$ values could be predicted based on corresponding EUL values as we proposed. 2) The EEKB prediction models were established with different $a$ values ($a=0.5$ and $a=2$) by using same 60 patients whose treatment plans were optimized by using mean dose (${EUD}^{a=1}$) as optimized objects in PAP modules. Thus, the $R^{2}$ of these EEKB predicted modules ($a=0.5$ and $a=2$) were reduced compared with the $R^{2}$ of the EEKB predicted modules with $a=1$. It is proved that, for maintaining high predicted accuracy and well linear correlation, the establishment of EEKB predicted modules with different $a$ values are required IMRT plans optimized with objects in same $a$ value as well.
